# Immunomodulatory effect of standardized *C. asiatica* extract on a promotion of regulatory T cells in rats

**DOI:** 10.1186/s12906-021-03394-z

**Published:** 2021-09-03

**Authors:** Supannikar Tawinwung, Dhirarin Junsaeng, Supanut Utthiya, Phisit Khemawoot

**Affiliations:** 1 Department of Pharmacology and Physiology, Faculty of Pharmaceutical Sciences, Chulalongkorn University, Bangkok, Thailand; 2 Department of Clinical Pharmacokinetics, Graduate School of Medical Sciences, Kanazawa University, Takara-machi, Kanazawa, Japan; 3 Chakri Naruebodindra Medical Institute, Faculty of Medicine Ramathibodi Hospital, Mahidol University, Bang Phli, Samut Prakarn, 10540 Thailand

## Abstract

**Background:**

ECa 233 is a standardized extract of *C. asiatica* containing the triterpenoid glycosides, madecassoside to asiaticoside in the ratio of (1.5 ± 0.5):1. Anti-inflammatory activities of ECa 233 have been reported; however the immunomodulatory effects of ECa 233 on regulatory T cells, which have a pivotal role in immune regulation, has not been elucidated. Therefore, we investigated the effects of ECa 233 on regulatory T cells that may provide benefits in autoimmune and chronic inflammatory diseases.

**Methods:**

ECa 233 was prepared as oral suspension in 0.5% carboxymethylcellulose and administered to male Wistar rats via oral gavage. The pharmacokinetics and toxicity of ECa 233 were evaluated. Splenic lymphocytes were isolated and analyzed by flow cytometry and qPCR to determine the immunomodulatory effects of ECa 233 on regulatory T cells.

**Results:**

All rats had good tolerability to ECa 233 and other test preparations. The pharmacokinetic study showed low oral bioavailability for both triterpenoids, with the maximum plasma concentration reached at 4 h for asiaticoside and at 0.5 h for madecassoside. Multiple oral administration of ECa 233 reduced the frequency of T cells, particularly CD8 T cells in rats. ECa 233 enhanced the percentage of regulatory T cells, characterized by high expression of CD25^+^ and upregulation of FoxP3 gene.

**Conclusions:**

The present study demonstrated that ECa 233 possesses immunosuppressive properties by enhancing regulatory T cells*.* These results provide in vivo evidence for the anti-inflammatory action of ECa 233, in line with previously reports, and the potential uses of ECa 233 in the treatment of chronic inflammatory and autoimmune diseases.

## Background

The immune system plays a critical role in the prevention of cancer and infectious diseases. However, dysregulation of the immune function is related to chronic inflammation and autoimmune diseases. To control immune homeostasis, multiple mechanisms are involved. Regulatory T cells (Tregs), a CD4 T-cell subset with immunosuppressive function, play a crucial role in regulating immune homeostasis and self-tolerance. Natural Tregs (nTregs) arise from the CD4^+^ thymocytes in the thymus [1], whereas peripheral naïve CD4 T cells can be differentiated into inducible Tregs in the presence of certain cytokines such as tumor growth factor-β (TGF-β) and interleukin-2 (IL-2) [2]. Tregs exhibit high surface expression of CD25 [3]. The transcription factor FOX head P3 (FoxP3) is a major regulator of the development and function of Tregs, in which mutation of the FoxP3 gene results in severe autoimmune disease [4]. Given the pivotal role of Tregs in immune regulation, adoptive transfer of Tregs provides therapeutic benefits in autoimmune and chronic inflammatory diseases such as multiple sclerosis, collagen-induced arthritis and type 1 diabetes [5–7]. In addition, several herbal medicines have been shown to reduce chronic inflammation and ameliorate autoimmune diseases by selective increases in Tregs [8–10].

*C. asiatica* (L.) Urban, the plant commonly found in tropical and temperate zones around the world, has been widely used in traditional medicine due to various health benefits, including learning and memory enhancement, wound healing, and anti-inflammation [11–14]. The active components of *C. asiatica* extracts are mainly triterpenoid glycosides madecassoside and asiaticoside [15]. To avoid variation in the compositions of the bioactive compounds, standardized extract ECa 233 was developed to control the amount of triterpenoid glycosides at > 80% with a constant of madecassoside to asiaticoside ratio of (1.5 ± 0.5) to 1 [16]. The standardized extract ECa 233 has been studied for its pharmacological activities in cognitive function [17, 18] and hepatoprotective effect [19]. Previous in vitro studies have also demonstrated that ECa 233 inhibits proinflammatory mediators and cytokines including ROS, NO, TNF-α, and IL-1β in LPS-stimulated macrophages and keratinocytes [20]. However, the effects of ECa 233 in immune modulation and Tregs generation have not been elucidated.

In our pharmacokinetic experiments of ECa 233 during 2016–2018, we consistently observed the accumulation of the two-triterpenoid glycosides in rat spleens with an approximate 10–100 fold of spleen to plasma ratio [21, 22], suggesting potential in vivo immunological effects of these compounds. This is also supported by traditional uses of *C. asiatica* in Thailand as an anti-inflammatory agent [23] and previous literatures showing immunomodulatory effects of alcoholic extracts of *C. asiatica* in rodents [24, 25]. In the present study, we aim to investigate in vivo immunological effects of ECa 233. The standardized extract ECa 233 was prepared as oral suspension and pharmacokinetic profiles as well as animal tolerability were also determined. Information obtained from this study will provide important evidence to confirm the ethnopharmacological knowledge of the use of the immunomodulatory actions of *C. Asiatica*.

## Methods

### Animals

Eight-week-old male Wistar rats were purchased from Nomura Siam International Co., Ltd., Bangkok, Thailand). Prior to the experiments, the rats were acclimatized for 2 weeks at a controlled temperature at 24 ± 2 °C and 40–60% humidity, a 12-h dark–light cycle with ad libitum access to food and water. All animal procedures were approved by the Institutional Animal Care and Use Committee of the Faculty of Pharmaceutical Sciences, Chulalongkorn University (approval no. 19–33-001, date of approval, April 1, 2019).

### Materials

Standardized extract ECa 233 powder was provided by Siam Herbal Innovation Co., Ltd., Bangkok, Thailand. The standardized extract contained 50% madecassoside and 40% asiaticoside, as determined by liquid chromatography tandem mass spectrometry (LC-MS/MS). Analytical standard asiaticoside (purity > 98.5%) was purchased from Sigma-Aldrich Corp, USA and madecassoside (purity > 96.7%) from Chromadex Corp, USA. Glycyrrhetinic acid (purity > 98.0%) as internal standard (IS) for LC-MS/MS analysis was purchased from Wako Pure Chemical Industries, Ltd., Japan. Dimethyl sulfoxide (DMSO) and carboxymethyl cellulose (CMC) used as a suspending agent were purchased from Sigma-Aldrich Corp., USA.

### Animal experiment

Rats, weighing more than 400 g, were put in metabolic cages and fasted overnight with free access to water before experiments. The animals were divided randomly into four groups (*n* = 6 in each) control, and treated with ECa 233,100 mg/kg via oral gavage, or with pure madecassoside 5.0 mg/kg or pure asiaticoside 4.0 mg/kg via intravenous administration. The intravenous preparations were freshly dissolved in 20% DMSO/NSS for injection. The oral preparations were freshly prepared by suspending the test compound in 0.5% CMC. To reduce pain and injury during drug administration and blood collection, rats were anesthetized with 5% isoflurane by chamber induction. Serial blood samples (300 μL) were collected at 0, 5, 15, and 30 min, and at 1, 2, 4, 8, and 24 h after dosing. Blood samples were centrifuged at 5000 *g* for 10 min, and plasma was collected and stored at − 20 °C until analysis. Determination of aspartate transaminase (AST), alanine transaminase (ALT), and creatinine levels were performed at pre-dosing, and at 0 h and 24 h after dosing. Creatinine level was determined by enzymatic Roche Cobas 6000 analyzer. In this enzymatic method creatinine is converted to creatine under the activity of creatininase. Creatine is then acted upon by creatinase to form sarcosine and urea. Sarcosine oxidase converts sarcosine to glycine and hydrogen peroxide, and the hydrogen peroxide reacts with chromophore in the presence of peroxidase to produce a color product that is measured at 546 nm (secondary wavelength = 700 nm). This is an endpoint reaction that agrees well with recognized HPLC methods, and it has the advantage over Jaffe picric acid-based methods that are susceptible to interferences from non-creatinine chromogens. For tissue collection, the rats was euthanized with isoflurane after oral dosing the test compound at 100 mg/kg for 7 days. After the dead was confirmed, spleen and blood were immediately collected, washed with normal saline solution, and send to immunology laboratory for further experiments. The control group received oral gavage of 0.5% CMC as vehicle control. All animal procedures were complied with the ethical principles and guidelines for the use of animals (National Research Council of Thailand, 2015).

### Determination of triterpenoids in biological samples

Protein precipitation by methanol was applied for preparation of LC-MS/MS samples. Briefly, plasma sample (50 μL) was mixed with 200 μL of methanol containing 10 ng of IS. The mixture was centrifuged at 10,000 *g* for 10 min and 10 μL of supernatant then injected for analysis by the LC-MS/MS system. Validation of the bioanalytical method of bioactive triterpenoid determination was based on previous study by our groups [26]. In brief, LC-MS/MS was performed on a Nexera ultra high-performance liquid chromatograph and 8060 triple quadrupole mass spectrometers (Shimadzu Co., Ltd., Japan). The stationary phase was Synergi Fusion-RP C18 column (Phenomenex Inc., USA) with 40 °C oven temperature. The mobile phase was 100% methanol and 0.2% formic acid in water with gradient elution. The flow rate was 0.5 mL/min and the volume of injection 10 μL. The analysis was conducted in a negative mode with mass-to-charge ratios of madecassoside, asiaticoside, and glycyrrhetinic acid of 973.40/503.30, 957.40/469.20, and 469.35/409.40, respectively.

### Splenocytes isolation

Spleens were harvested from rats administered with ECa 233 or vehicle control (control group) and prepared in an aseptic condition. Briefly, the spleens were minced and mashed through a cell strainer (100 μm) using a 5-mL syringe to obtain a homogeneous cell suspension. The contaminating erythrocytes were lysed using 1X Red Blood Cell lysis buffer (BioLegend, USA). The cell pellets were washed twice with PBS and resuspended in complete medium (Roswell Park Memorial Institute (RPMI) 1640 Medium containing 10% fetal bovine serum (FBS) for further analysis. The viability of splenocytes was determined using trypan blue dye with a result of more than 95% viability.

### Flow cytometry

Freshly isolated splenocytes were washed and resuspended in PBS containing 1% FBS. The cell pellets were incubated with fluorochrome-conjugated antibodies for 20 min at 4 °C and washed twice with PBS (1% FBS). The following monoclonal antibodies were used: PE anti-rat CD45RA antibody, APC anti-rat CD3 antibody, PEcy7 anti-rat CD antibody, PE anti-rat CD8 antibody, and FITC anti-rat CD25 antibody. All antibodies were obtained from BioLegend, USA. Samples were acquired and analyzed with BD Accuri C6 Plus Flow Cytometer (BD Biosciences, USA).

### Real time RT-PCR

Total RNA was extracted from the frozen spleen sections using RNeasy Mini Kit (Qiagen, Germany) and converted into cDNA using QuantiTect Reverse Transcription Kit (Qiagen, Germany). The synthesized cDNA was used as the template to determine the relative expression of immune related genes including *Ifng, Il2, Il17a, Il10, Foxp3, Tgfb*. The quantitative RT-PCR was performed with a StepOne plus RT-PCR using Lunar Universal qPCR Master Mix (New England BioLabs, USA). The mRNA expression of candidate genes was normalized to the expression level of β-actin and presented as relative quantification using the calculation of 2^-ΔΔCt^. The primers used in the experiments are listed in the table below:

**Table:** 

Gene	Forward (5′-3′)	Reverse (5′-3′)
IFN-γ	GCCCTCTCTGGCTGTTACTG	CCAAGAGGAGGCTCTTTCCT
IL-2	AAACTCCCCATGATGCTCAC	GAAATTTCCAGCGTCTTCCA
IL-17A	CCATCCATGTGCCTGATGCT	AAGTTATTGGCCTCGGCGTT
IL-10	CGACGCTGTCATCGATTTCTC	CAGTAGATGCCGGGTGGTTC
FoxP3	TCATGGGCCCTCAAAGTTAC	GTGTGGTTTTCTGGGATGCT
TGFβ	CTTTGTACAACAGCACCCGC	TAGATTGCGTTGTTGCGGTC

### Proliferation assay

Freshly isolated splenocytes were resuspened in RPMI 1640 culture media containing 10% FBS and seeded in 96-well plates at 5 × 10^4^ cells per well. The splenocytes were then stimulated with either 5 mg/mL of LPS (Sigma-Aldrich, USA) or 5 mg/mL of Concanavalin A (Sigma-Aldrich, USA) for 72 h at 37 °C in 5% CO_2_. The cell viability was assessed using the WST-1 assay (Roche Diagnostics, Switzerland) according to the manufacturer’s instructions.

### Pharmacokinetic and statistical analyses

All pharmacokinetic parameters were computed by non-compartmental analysis using PK Solution 2.0 software (Summit Research Service, USA). The pharmacokinetic parameters were reported as maximal plasma concentration (C_max_), time to reach maximal plasma concentration (T_max_), area under the curve from time 0 to 24 h (AUC_0-t_), area under the curve from time 0 to infinity (AUC_0-inf_), mean resident time (MRT), volume of distribution (Vd), total clearance (CL), and elimination half-life (T_1/2_). The absolute oral bioavailability was calculated as (AUCp.o. ÷ dose p.o.) ÷ (AUCi.v. ÷ dose i.v.). Pharmacokinetic data are presented as mean ± standard deviation. Blood chemical parameters were analyzed by Mann–Whitney U-test to compare the significance of differences between pre-dosing and 24 h post-dosing, with a *p*-value of less than 0.05. All statistical analyses were calculated using SPSS version 16 (SPSS, Inc., USA). Pharmacodynamic results are presented as mean ± standard error of mean (SEM). The data were analyzed with an unpaired Student’s *t*-test. A *p*-value of < 0.05 was considered statistically significant.

## Results

### In vivo tolerability of standardized extract ECa 233

**Fig. 1:**
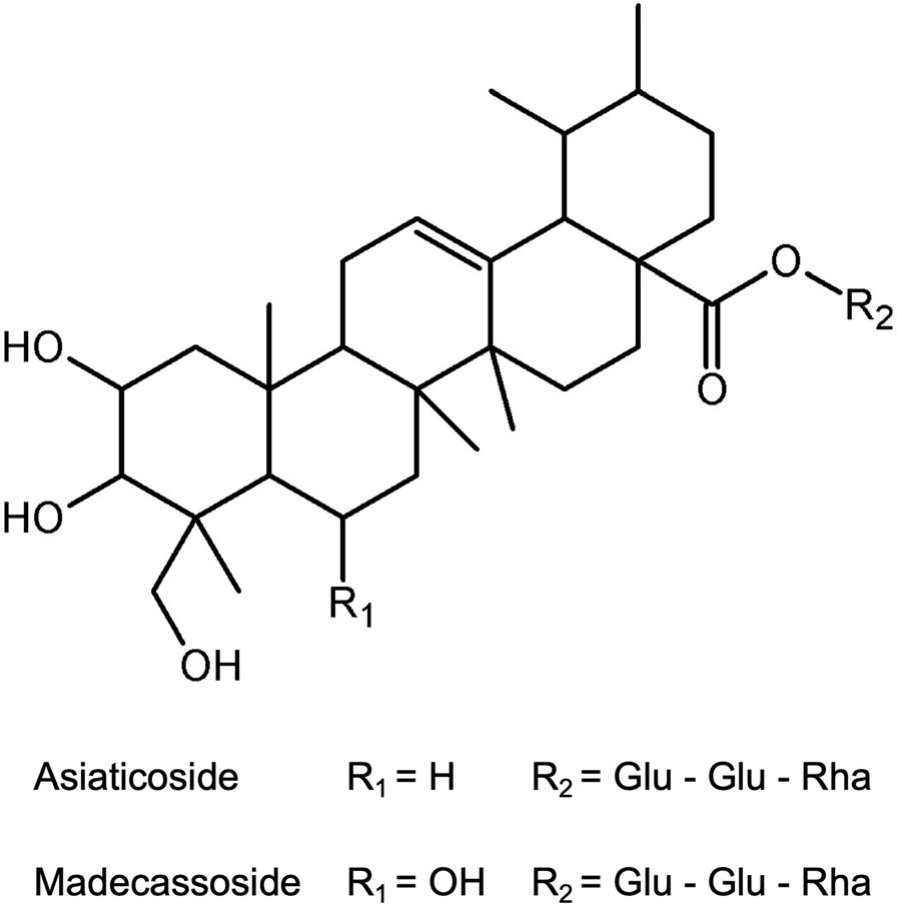
Chemical structure of triterpenes from *Centella asiatica* (Glu: glucose, Rha: rhamnose)

In order to study the immunomodulatory effects of ECa 233 in vivo, we first evaluated the tolerability and pharmacokinetics of standardized extract ECa 233. Figure 1 illustrates the molecular structures of the bioactive triterpenoid glycosides in ECa 233, including asiaticoside and madecassoside. The doses of pure triterpenoid glycosides were calculated based on the percentage labeled amount of ECa 233, as mentioned in Materials and Methods. Table 1 shows the result of tolerability after the administration of the compounds for 24 h. The animals presented good tolerability with normal physical appearance. The levels of liver enzymes, including AST and ALT, were also within the normal range and no significant differences were observed between ECa 233 and the pure compounds, or between before and after administration. In addition, the animals exhibited normal kidney function as there was no significant alteration in creatinine levels after treatment.

**Table 1:** Physical appearance and biochemical profiles of pre-dose vs. post-dose. Data are shown as mean ± SD (*n* = 6; *P < 0.05)

Compounds	Route	Physical appearance		Creatinine (mg/dL)		AST (U/L)		ALT (U/L)	
		Pre-dose (0 h)	Post-dose (24 h)	Pre-dose (0 h)	Post-dose (24 h)	Pre-dose (0 h)	Post-dose (24 h)	Pre-dose (0 h)	Post-dose (24 h)
Asiaticoside	IV 4 mg/kg	Normal	Normal	0.60 ± 0.03	0.61 ± 0.04	42.40 ± 14.54	54.80 ± 9.20	26.80 ± 3.83	27.40 ± 5.32
Madecassoside	IV 5 mg/kg	Normal	Normal	0.64 ± 0.04	0.67 ± 0.05	46.20 ± 4.87	63.20 ± 16.81	23.80 ± 2.59	24.00 ± 4.85
ECa 233	PO 100 mg/kg	Normal	Normal	0.61 ± 0.02	0.64 ± 0.01	45.02 ± 7.12	61.80 ± 15.69	24.80 ± 4.15	27.60 ± 6.11

### Pharmacokinetics of standardized extract ECa 233

**Fig. 2:**
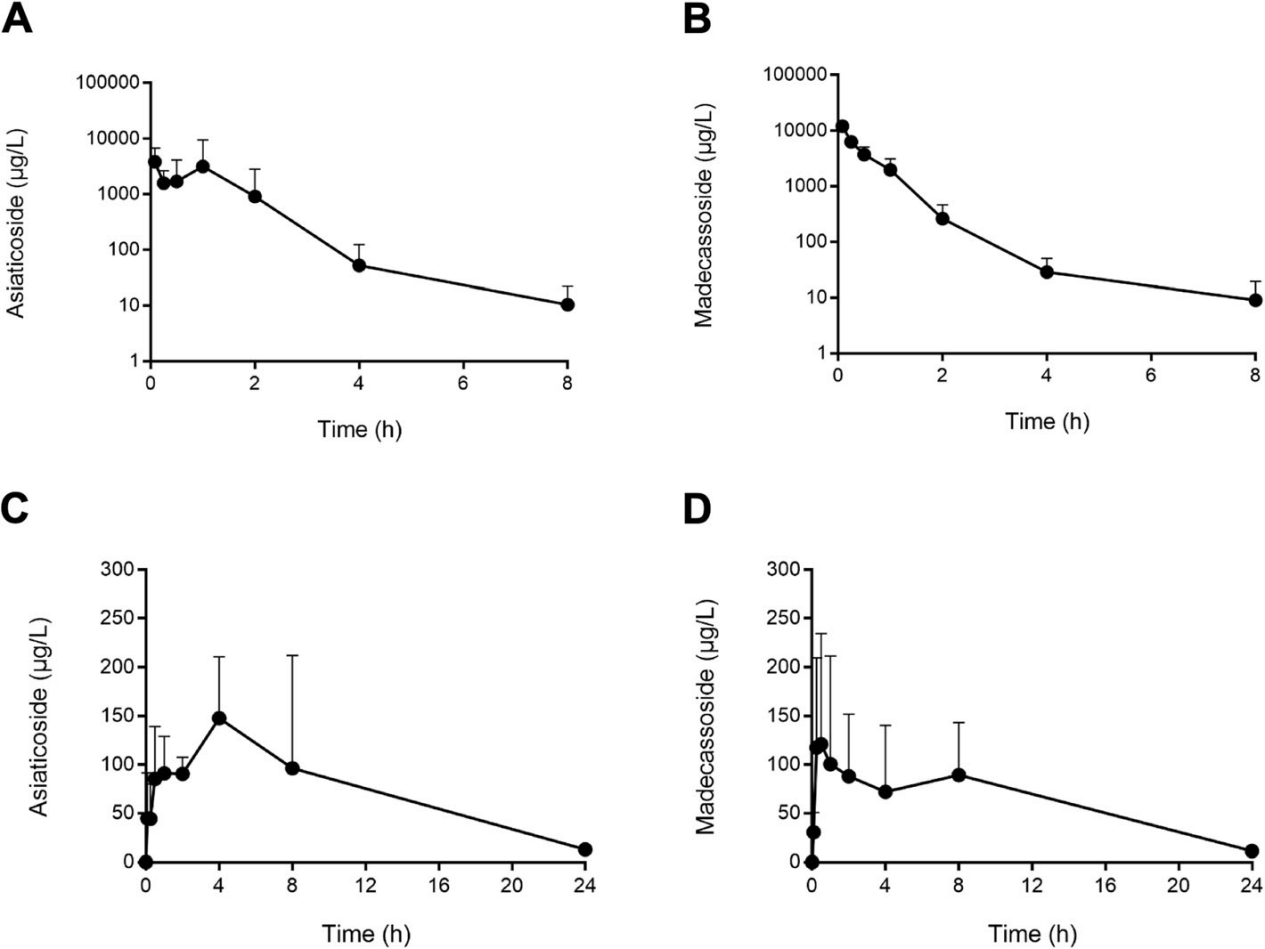
Plasma concentration–time profiles of madecassoside and asiaticoside following the intravenous administration (**A**, **B**) and oral administration (**C**, **D**) of ECa 233

We next analyzed the plasma concentration–time profile of oral ECa 233. The mean plasma concentrations of asiaticoside and madecassoside were analyzed over time after the administration of ECa 233 or the pure compounds, as shown in Fig. 2. Intravenous injection of pure asiaticoside or madecassoside showed maximum plasma levels of approximately 3000 μg/L and 10,000 μg/L, respectively. The plasma concentrations decreased gradually to approximately 10 μg/L at 8 h after dosing (Fig. 2a and b). The plasma concentration–time profile of oral administration of ECa 233 also exhibited a rapid gastrointestinal absorption with the detection of both triterpenoid glycosides within 5 min after oral dosing. However, the maximum plasma levels (C_max_) of asiaticoside and madecassoside after oral administration of ECa 233 were 147.84 ± 62.82 μg/L and 121.24 ± 113.32 μg/L, respectively. The T_max_ was reached at 4 h for asiaticoside and at 0.5 h for madecassoside (Fig. 2c and d). The complete pharmacokinetics parameters derived from non-compartmental analysis of concentration–time profile after oral administration of ECa 233 and pure compounds are reported in Table 2. The absolute oral bioavailability of ECa 233 was 2.36% for asiaticoside and 1.58% for madecassoside.

**Table 2:** Pharmacokinetic parameters of madecassoside and asiaticoside, a main component of ECa 233. Data are shown as mean ± SD (*n* = 6)

Parameters	Asiaticoside		Madecassoside	
	Asiaticoside 4 mg/kg IV	ECa 233 100 mg/kg PO	Madecassoside 5 mg/kg IV	ECa 233 100 mg/kg PO
C_max_ (μg/L)	N/A	147.84 ± 62.82	N/A	121.24 ± 113.32
T_max_ (h)	N/A	4.00 ± 2.45	N/A	0.50 ± 1.59
AUC_0-t_ (μg.h/L)	7588.69 ± 12,144.79	1591.66 ± 1430.06	8575.63 ± 3238.74	1307.35 ± 878.01
AUC_0-inf_ (μg.h/L)	7605.43 ± 12,142.80	1797.82 ± 1324.85	8691.56 ± 3330.53	1377.78 ± 882.71
MRT (h)	0.74 ± 0.31	8.80 ± 4.85	0.65 ± 0.18	6.41 ± 3.56
Vd (L/kg)	3.27 ± 3.91	284.78 ± 229.02	3.25 ± 2.22	189.68 ± 120.78
CL (L/h/kg)	2.25 ± 1.66	35.48 ± 26.99	0.67 ± 0.31	57.83.17 ± 47.04
T_1/2_ (h)	0.98 ± 0.56	6.48 ± 6.18	4.50 ± 3.76	3.49 ± 2.19
Bioavailability (%)	N/A	2.36	N/A	1.58

### Effects of ECa 233 on splenic lymphocytes

**Fig. 3:**
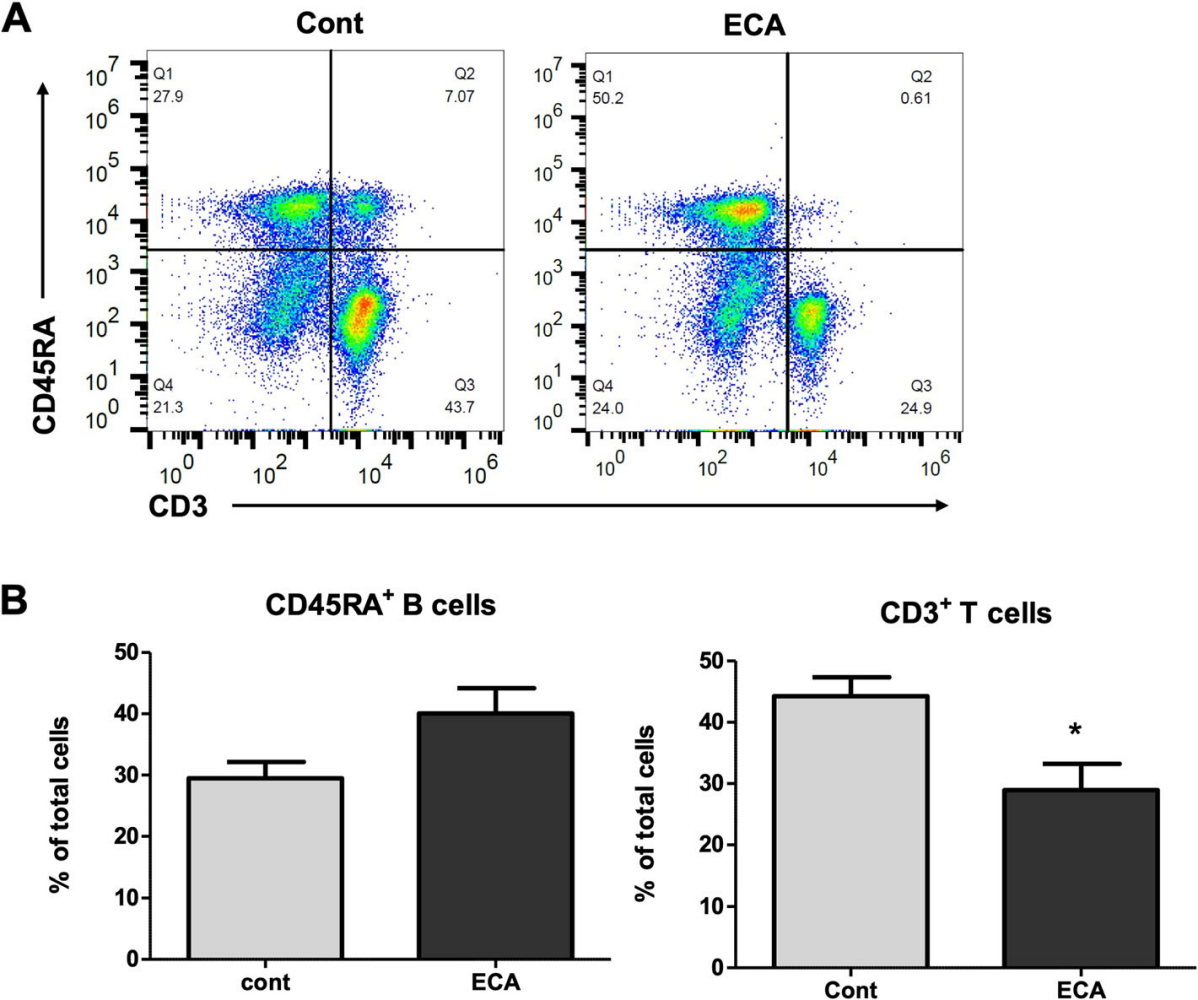
Splenic lymphocyte subsets from rats treated with ECa 233 or vehicle control (**A**) flow cytometry representative dot plots (**B**) Bar graphs show the percentage of CD45RA^+^ cells and CD3^+^ cells, Data are shown as mean ± SE (*n* = 6; *P < 0.05)

**Fig. 4:**
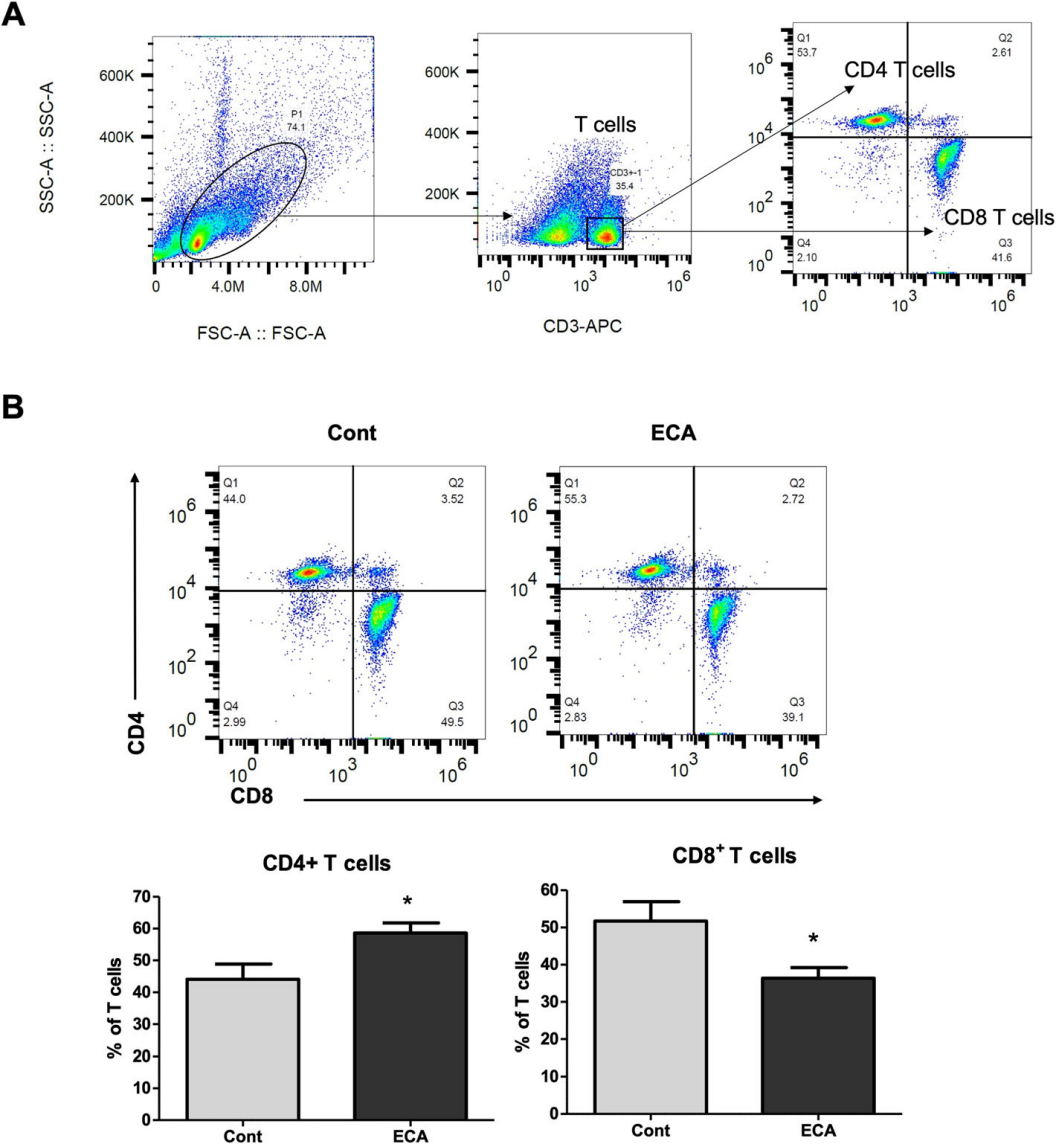
Evaluation of CD4 and CD8 T cells from the splenocytes of rats received ECa 233 via oral administration (**A**) Gating strategy for the percentage of CD4 and CD8 T cells from splenocytes. **B** Dot plots represent the splenic CD4 and CD8 T cells from control and ECa 233 groups. Bar graphs show the percentage of CD4 T cells and CD8 T cells from rats treated with ECa 233 vs vehicle control, Data are shown as mean ± SE (*n* = 6; *P < 0.05)

In the present study, we sought to investigate the effects of ECa 233 on splenic lymphocytes. We first examined the proliferative responses of splenocytes obtained from rats treated with ECa 233. The splenocytes were isolated and freshly stimulated with Con A or LPS. The proliferative response was measured by using WST-1 assay. We observed no significant differences on the mitogenic responses of splenocytes between the control and ECa 233-treated groups. We next analyzed the subsets of splenocytes from rats treated with ECa 233. As shown in Fig. 3a and b, the percentage of CD45RA^+^ B cells was increased in the splenocytes of rats receiving ECa 233 treatment compared to the vehicle group, but this was not statistically significant. However, ECa 233 significantly reduced the proportion of CD3^+^ T cells in the rat splenocytes (38 ± 1.9 vs 44 ± 3.1, ECa 233 vs control, *p* < 0.05). Further phenotypic analyses of T-cell subsets showed that oral administration of ECa 233 increased the percentage of CD4^+^ T cells and reduced the percentage of CD8^+^ T cells in the spleen (Fig. 4a and b), resulting in a higher ratio of CD4 to CD8 with ECa 233 treatment. These results suggested that ECa 233 modulates cellular immunity in vivo*.*

### ECa 233 enhances the frequency of splenic regulatory T cells

**Fig. 5:**
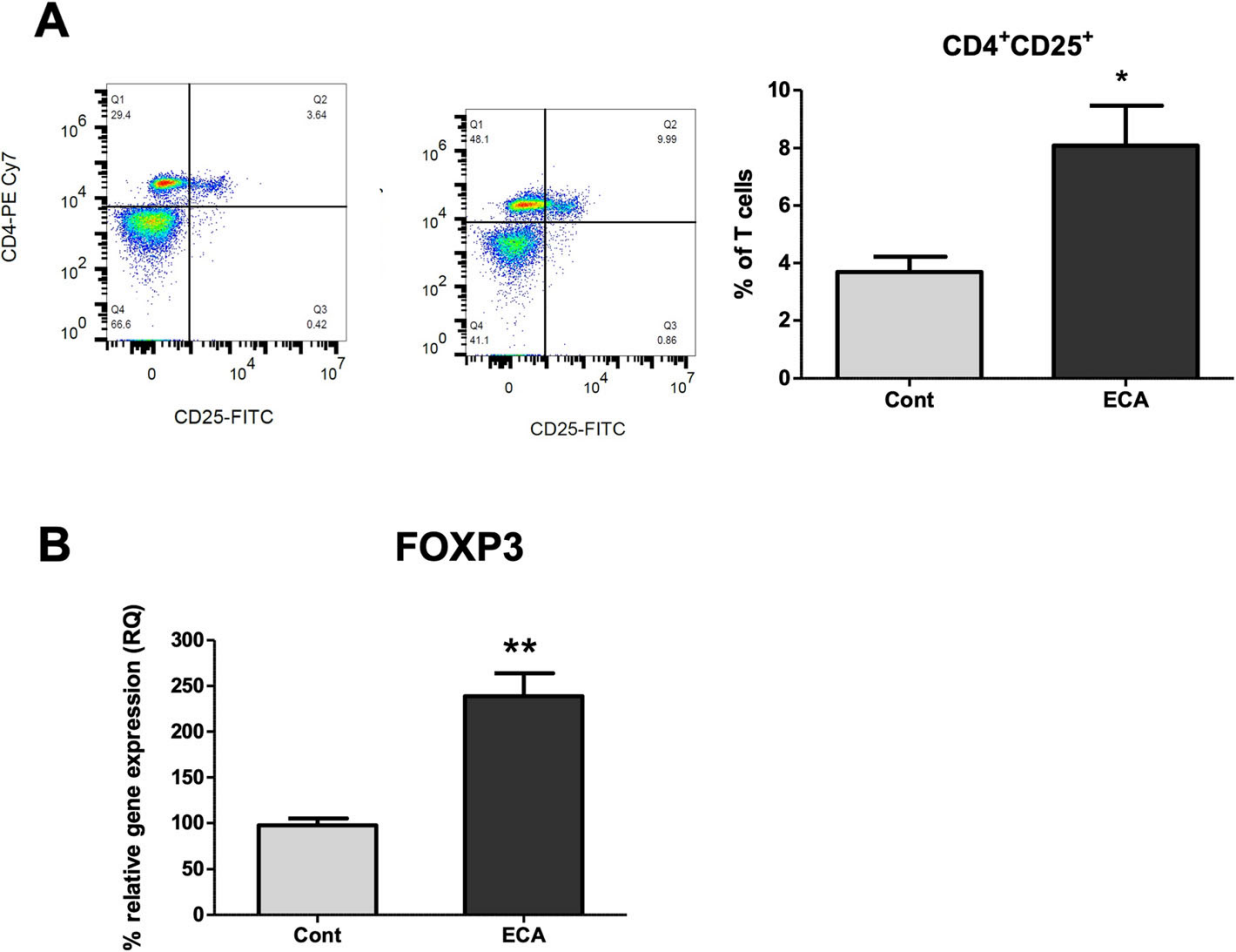
Effect of ECa 233 on the splenic regulatory T cells (**A**) Percentage of T cells expressing CD4 ^+^ CD25^+^ (**B**) The mRNA expression of FoxP3 from the spleen of rats. Data are shown as mean ± SE (*P < 0.05, **P < 0.01)

As an increase in the proportion of splenic CD4^+^ T cells with ECa 233 treatment was observed, we next investigated the effects of ECa 233 on the frequency of regulatory T cells (Tregs). Previous studies have shown that unstimulated CD4^+^ T cells expressing CD25 exhibit a suppressive activity; thus, we analyzed the percentage of CD4^+^CD25^+^ Tregs in the spleen. In this study, we observed that oral administration of ECa 233 significantly increased the percentage of CD4^+^CD25^+^ T cells in the splenocytes compared to the control group (Fig. 5a). In addition, the mRNA expression of splenic FoxP3, a major transcription factor regulating Tregs, was upregulated with the in vivo treatment of ECa 233 (Fig. 5b). These data suggest that oral administration of ECa 233 increases the population of immunosuppressive Tregs in the spleen of rats.

### ECa 233 upregulates transcriptions of Treg-derived cytokines

**Fig. 6:**
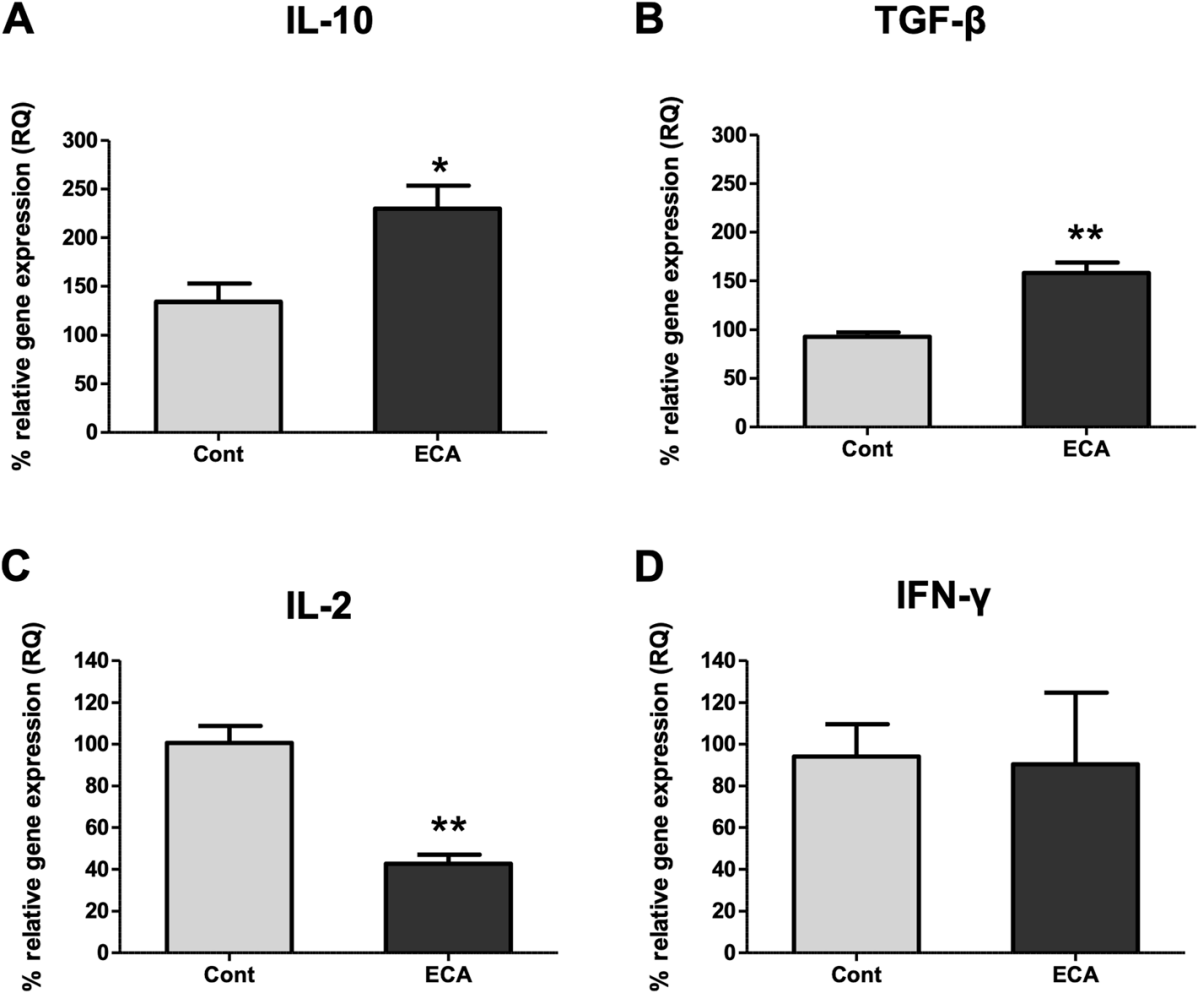
Effects of ECa 233 on inflammatory cytokines. Total RNA were isolated from spleen tissues obtained from rats treated with ECa233 or vehicle control. The mRNA expression of IL-10, TGF-β, IL-2, IFN-γ were analyzed by real time RT-PCR. Data are shown as mean ± SE (*P < 0.05, **P < 0.01)

Tregs have been known to secrete anti-inflammatory mediators, including IL-10 and TGF-β. Therefore, we measured transcription of these immunosuppressive cytokines in the spleen after oral administration of ECa 233. There was a significant increase in mRNA expression level of IL-10 (Fig. 6a) and TGF-β (Fig. 6b) with ECa 233 treatment. Additionally, the mRNA expression of T-cell growth factor IL-2 was reduced in the spleen of ECa 233-treated rats, while there was no difference in IFN-γ gene expression between the two groups (Fig. 6c and d).

## Discussion

Standardized extract ECa 233 is a water-insoluble extract of *C. asiatica* that contains not less than 80% triterpenoid glycosides with an asiaticoside/madecassoside ratio of (1.5 ± 0.5):1 [16]. The pharmacological effects of the extract have been evidenced to include wound healing, anti-inflammation, and cognitive function enhancement. Our group has previously reported a comprehensive pharmacokinetic study of standardized extract ECa 233 in which the administration of oral ECa 233 resulted in a high plasma concentration and long half-life of triterpenoid glycosides compared with the oral administration of pure compounds in equivalent doses [21, 22]. In the same study, a significant amount of madecassoside and asiaticoside in the spleen was reported, ranging from 2000 to 4000 ng/g of tissue and approximately 10-fold higher on day 7 vs day 1 after dosing [21]. Together with its anti-inflammatory effects and its accumulation in spleen, these prompted us to investigate the immunodulatory effects of ECa 233 in vivo after oral administration.

In the present study, ECa 233 was prepared as oral suspension in 0.5% CMC vehicle and administered to the animals via oral gavage. CMC is known to be a pharmacologically inert vehicle for oral suspension that provides a good safety profile with no damage to intestinal integrity [27]. The safety of ECa 233 oral suspension was confirmed in this study and the results coincide with a previous acute toxicity study showing that the 50% lethal dose of ECa 233 was > 10 g/kg in rodents [28]. Here, we also report that after administration of ECa 233 oral suspension the C_max_ was approximately 120–140 μg/L with values of T_max_ of 0.5 h and 4 h for madecassoside and asiaticoside, respectively. These pharmacokinetic parameters are lower than reported in our previous study using 50% DMSO in NSS as vehicle. This is possibly due to the high solubility of the DMSO preparation, resulting in fast absorption. However, the low bioavailability of ECa 233 is similarly observed in this study due to the large molecular structure of triterpenoid glycosides. In addition, madecassoside is shown to be a substrate for P-glycoprotein and multidrug-resistant protein 2, affecting the level of absorbed compound [29].

In this study, we also determined the effect of ECa 233 on the immune system and showed for the first time that oral administration of ECa 233 reduces the frequency of T cells, particularly CD8 T cells in splenocytes, suggesting an immunosuppressant property of the extract. We also found a reduced splenic IL-2 gene expression, in consistent with the reduction in T cells. IL-2 is known to play a critical role in T cell survival and proliferation [30]. Interestingly, IFN-γ gene expression was not altered after ECa 233 administration, suggesting that the TH1 immunity may not be affected by the treatment. More importantly, we report an increase in CD4^+^CD25^+^ T cells and upregulation of FoxP3 gene in the spleen after in vivo treatment of ECa 233. A previous study has shown that mRNA expression of FoxP3 and inhibitory molecules, including IL-10, Lag3, and CTLA-4, are highly upregulated in unstimulated rat CD4^+^CD25^+^ T cells. In addition, adoptive transfer of these cell subsets prevent insulitis induced by diabetogenic T cells in rats, suggesting an immunoregulatory role of this cell subset [31].

Triterpenoid glycosides have been shown to exhibit collective pharmacological properties and modulate multiple molecular targets including genes, signaling molecules, receptors and cellular proteins. Previous studies suggest that *C. asiatica* exerts its an anti-inflammatory action through suppression of pro-inflammatory cytokines, chemokines and adhesion molecules [32]. For example, Luo et al. reported that madecassoside suppressed TNF-α, IL-1β and IL-6 secretion induced by oxygen-glucose deprivation/reperfusion-induced injury in microgial cells, mediated by inhibition of TLR4 and its downstream signaling molecules MyD88/NF-κB [33]. In silico docking study of triterpenoid also revealed the IKKβ inhibition and downstream NF-κB inhibition as the major mechanism of anti-inflammatory action of asiatic acid [34]. For the direct effect on immune cells, the triterpenoid fraction of *C. asiatica*, madecassic acid, were shown to promote differentiation of Tregs and attenuate dextran sulfate sodium-induced colitis in mice via regulating the PPARγ/AMPK/ACC1 pathway [35]. Similarly, oral administration of triterpenoid saponin, madecassoside provided anti-arthritis effects through enhanced secretion of IL-10 from the small intestine of collagen induced arthritis rats and the accumulation of Foxp3^+^ cells in the lamina propria [36]. Consistently, our present data demonstrate that oral administration of *C. asiatica* standardized extract ECa 233 promotes the induction of splenic CD4^+^CD25^+^ Treg cells and anti-inflammatory cytokine genes IL-10 and TGF-β thus supporting the potential therapeutic benefits of *C. asiatica* standardized extract ECa 233 in chronic inflammatory and autoimmune-related diseases.

## Conclusions

The present study investigated the in vivo immunomodulatory effects of a standardized extract of *C. asiatica*. Oral administration of ECa 233 provided safety and conformity of pharmacokinetic profiles. The results demonstrated that ECa 233 enhanced the frequency of regulatory T cells and their associated anti-inflammatory cytokines. Moreover, cytotoxic T cells and interleukin-2 were reduced with ECa 233 treatment. Our findings confirm the immunosuppressive activity of ECa 233 and support a further study to develop ECa 233 as a promising candidate for clinical use in chronic inflammatory and autoimmune diseases.

## Data Availability

The datasets used and/or analysed during the current study are available from the corresponding author on reasonable request.

## Abbreviations

Tregs: Regulatory T cells
FoxP3: Forkhead box protein P3
ROS: Reactive oxygen Species
NO: Nitric Oxide
TNF-α: Tumor necrosis factor- α
TGF-β: Transforming growth factor- β
IL-1β: Interleukin-1β
IL-10: Interleukin-10
LPS: Lipopolysaccharide
IL-2: Interleukin-2
ConA: Concanavalin A
CMC: Carboxymethylcellulose
